# A Case of Invasive Ductal Carcinoma with Axillary Skip Metastasis Confined to the Interpectoral (Rotter’s) Lymph Node

**DOI:** 10.70352/scrj.cr.25-0590

**Published:** 2026-01-27

**Authors:** Emiri Sugiyama, Rina Suzuki, Jin Takano, Yasuharu Tokuyama, Akemi Morikawa, Kousuke Nishimura, Kazuhiro Ishihara

**Affiliations:** Department of Surgery, Gihoku Kosei Hospital, Yamagata, Gifu, Japan

**Keywords:** axillary skip metastasis, interpectoral lymph node, Rotter’s node, breast cancer, sentinel lymph node biopsy

## Abstract

**INTRODUCTION:**

Axillary skip metastasis is a rare phenomenon in breast cancer and is defined as metastasis to level II or III lymph nodes without involvement of level I nodes. Interpectoral (Rotter’s) nodes are situated between the pectoralis major and minor muscles and may occasionally be overlooked during sentinel node (SN) mapping. Reports of isolated interpectoral node metastases are rare. Here, we present a unique case of breast cancer with isolated interpectoral node involvement despite a negative sentinel lymph node, underscoring the clinical implications of preoperative imaging and surgical planning.

**CASE PRESENTATION:**

A 69-year-old woman was referred to our hospital after an abnormality was detected by mammography. MRI demonstrated a 20-mm enhancing breast mass located in the deep portion of the upper outer quadrant, along with a strongly enhancing 6-mm interpectoral lymph node; no suspicious axillary level I nodes were identified. The patient underwent a mastectomy with sentinel and interpectoral node biopsies. The SN was negative, whereas the interpectoral node was positive, prompting axillary dissection. Histology confirmed a 15-mm invasive ductal carcinoma, with only the interpectoral node being positive among the 12 dissected nodes. Immunohistochemistry showed an ER-positive, PgR-positive, and HER2-negative status. The patient was started on adjuvant endocrine therapy. Her postoperative course was uneventful and she remained disease-free at 54 months of follow-up.

**CONCLUSIONS:**

This extraordinarily rare case of axillary skip metastasis limited to the interpectoral node emphasizes the potential for false-negative SN biopsies. Careful review of preoperative images, particularly MRI images, is crucial to avoid understaging. Awareness of interpectoral node involvement may help guide appropriate treatment strategies for selected patients.

## Abbreviations

ER
: estrogen receptor
HER2
: human epidermal growth factor receptor 2
PgR
: progesterone receptor
PMRT
: postmastectomy radiotherapy
SN
: sentinel node
SPECT
: single photon emission computed tomography

## INTRODUCTION

Axillary skip metastasis occurs when breast cancer spreads directly to level II or III lymph nodes without the involvement of level I nodes. Its incidence is low (0.3%–1.6%). Interpectoral node metastasis without axillary involvement is rare. Herein, we report a case and briefly review its clinical implications.

## CASE PRESENTATION

**Fig. 1:**
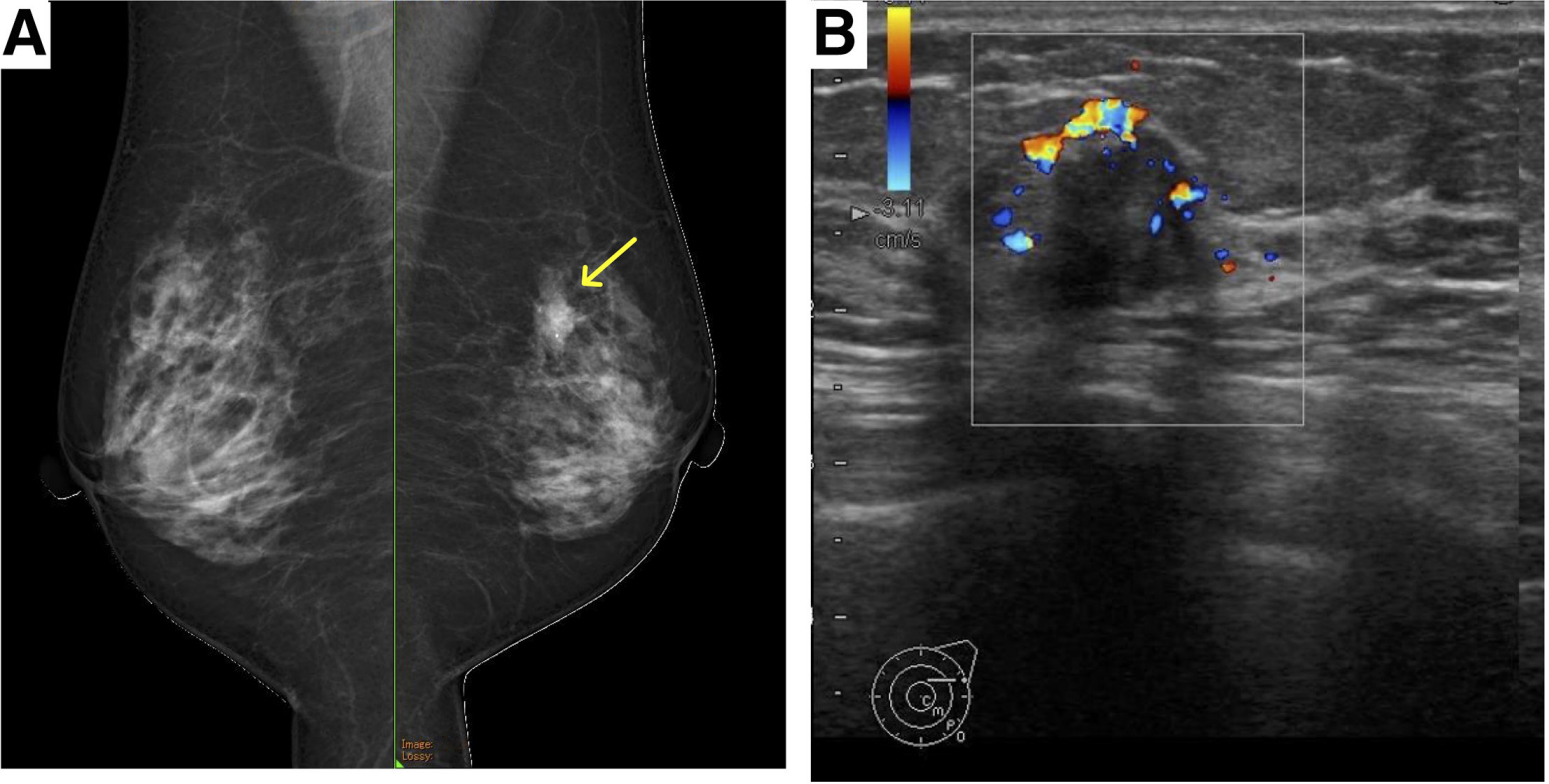
Mammography and ultrasonography. (**A**) Mammography (mediolateral oblique view) shows a mass with indistinct margins accompanied by microcalcifications in the left medial-outer (M–O) region (arrow). (**B**) Ultrasonography reveals an irregular, hypoechoic mass with a coarse but well-defined margin, measuring 16 × 16 × 11 mm, in the left central (C) region.

**Fig. 2:**
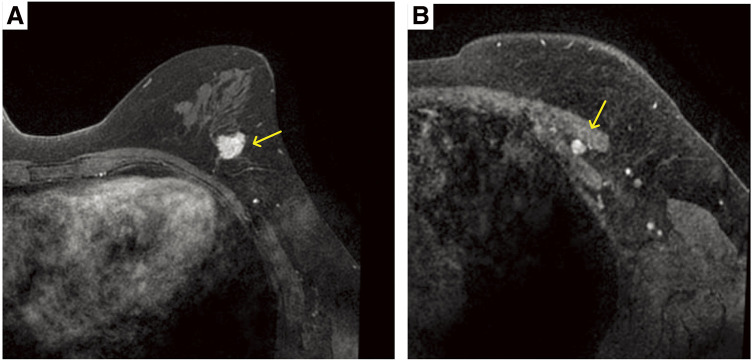
Contrast-enhanced breast MRI. (**A**) A mass in the left central (C) region showing rapid washout enhancement (arrow). (**B**) An enlarged, oval interpectoral lymph node with marked enhancement (arrow), suspicious for metastasis.

A 69-year-old woman presented to our hospital with an abnormal screening mammogram. Physical examination revealed a firm, immobile, 20-mm mass in the upper outer quadrant of the left breast. Mammography revealed an ill-defined mass with microcalcifications (**Fig. 1A**). Ultrasonography revealed a 16 × 16 × 11-mm irregular, hypoechoic lesion (**Fig. 1B**). MRI revealed a 20-mm rapidly enhancing lesion and a 6-mm enlarged interpectoral lymph node showing strong enhancement (**Fig. 2**). The interpectoral node could not be visualized on targeted axillary ultrasonography, and fine-needle aspiration was not performed. Although MRI suggested possible involvement of the interpectoral node, the finding was regarded as suspicious rather than definitive, and the preoperative clinical stage was therefore determined to be cN0. CT showed no distant metastasis. Core needle biopsy indicated invasive ductal carcinoma (tubule-forming type), which was ER- and PgR-positive, HER2-negative (1+), with a Ki-67 index of 2%–7%.

**Fig. 3:**
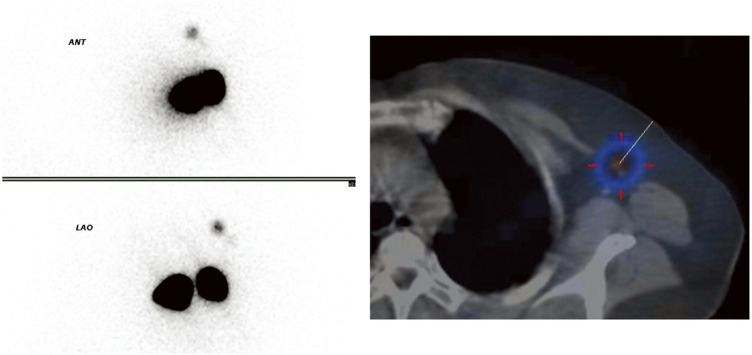
Sentinel lymphoscintigraphy. Only 1 lymph node at level I in the axilla is visualized.

The patient was diagnosed with left-sided breast cancer (cT1N0M0, Stage I). The patient underwent mastectomy with sentinel and interpectoral lymph node biopsies. SN mapping was performed using a dual-tracer technique (99mTc-phytate + indocyanine green). Subdermal injections of 99mTc-phytate were administered into the subdermal tissue directly over the tumor and the periareolar subdermal tissue (0.5 mL each, total activity ~0.5 mCi), and planar scintigraphy followed by SPECT/CT was performed approximately 4 hours after injection. Indocyanine green (1 mL at each site) was injected immediately before surgery at the same 2 locations. Both tracers demonstrated uptake in a single level I lymph node (**Fig. 3**). During surgery, the SN was identified (probe count, 561) and was found to be negative in intraoperative frozen sections. However, the interpectoral lymph nodes were positive, and axillary lymph node dissection up to levels I and II was subsequently performed.

**Fig. 4:**
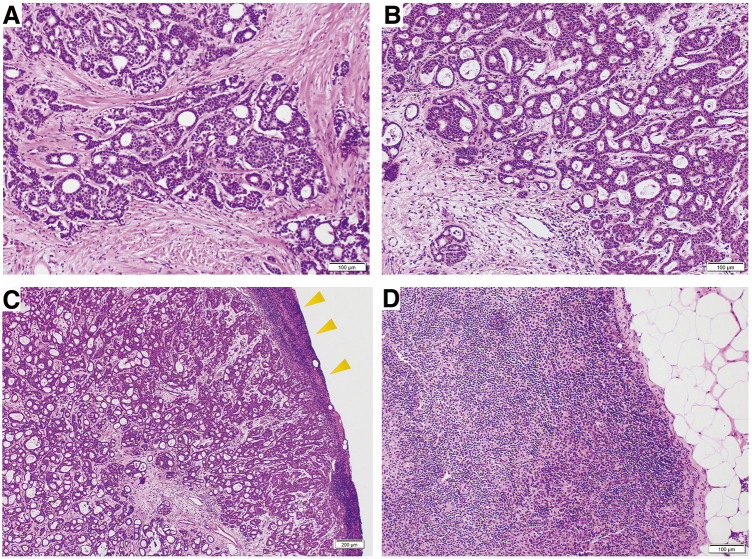
Surgical specimen, hematoxylin and eosin (H&E) staining. (**A**) Primary tumor: tumor cells are evident. Scale bar = 100 μm. (**B**) Interpectoral lymph node: tumor cells, resembling those of the primary lesion, extensively involve the node. Scale bar = 100 μm. (**C**) Interpectoral lymph node: tumor cells extensively involve the node, with small residual areas of sinus and lymphoid tissue preserved at the periphery (arrowheads). Scale bar = 200 μm. (**D**) Sentinel lymph node: no tumor cells are identified. Scale bar = 100 μm.

The final pathological examination revealed an invasive ductal carcinoma (15 mm) with no lymphovascular invasion. Of the 12 dissected nodes, only the interpectoral nodes were positive. Pathological examination of the interpectoral node showed metastatic carcinoma involving a substantial portion of the node; however, small portions of lymphoid tissue and sinus architecture were still identifiable, indicating that the node was not completely replaced by the tumor (**Fig. 4**). The final stage was pT1pN1a (SN 0/1, level I/II 0/12, interpectoral 1/1) M0, stage IIA. The patient recovered well, was discharged on POD 7, and has remained disease-free for 54 months while on aromatase inhibitor therapy. PMRT was not performed.

## DISCUSSION

Axillary skip metastasis, defined as metastasis to level II or III lymph nodes without involvement of level I nodes, is uncommon and reported in only 0.3%–1.6% of breast cancers.^1–3)^ Isolated interpectoral node involvement is rare. Cody et al. documented that only 0.5% of patients with axillary node-negative disease harbored metastasis in the interpectoral node, which is occasionally the sole site of nodal disease.^2)^ Haagensen et al. also reported interpectoral node metastases in 8.7% of radical mastectomy specimens, although true skip metastases confined to this node were less frequent.^3)^

Recent large-scale studies have confirmed the rarity of isolated interpectoral metastases and complexity of axillary staging. In the *American Journal of Roentgenology* cohort of 1300 breast cancers, staging nodal ultrasound with fine-needle aspiration detected skip metastases in 2.6% of all cases and 7.2% of node-positive cases, most frequently to the supraclavicular or level III nodes, with an over-representation of lobular and mixed histologies.^4)^ Yan et al. emphasized that interpectoral node metastasis generally reflects advanced axillary burden rather than an isolated disease.^5)^ Similarly, Keelara et al. observed interpectoral nodes in 35.7% of axillary dissections and metastases in 27.3% of cases; however, no isolated cases have been reported.^6)^ These findings underscore that isolated interpectoral node metastases are uncommon in modern clinical studies.

However, rare case reports have demonstrated isolated involvement of nodes. Ishige described recurrence limited to the interpectoral node 8 years after breast cancer surgery despite a negative SN biopsy,^7)^ whereas Matsuo et al. reported a case in which the interpectoral node was identified as the only metastatic site on preoperative imaging.^8)^ Our case adds to the literature by documenting MRI-detected, SN-mapping-missed, and isolated interpectoral node metastases.

The mechanism of skip metastasis can be explained by anatomical variations in the lymphatic flow. Previous anatomical and lymphoscintigraphic studies have demonstrated alternative drainage routes, including channels traversing or perforating the pectoralis major muscle, which allow direct flow to the interpectoral node while bypassing level I nodes.^1,2,5,9)^

Anatomical studies have demonstrated an alternative deep lymphatic pathway running through or along the pectoralis major muscle toward the interpectoral region, independent of superficial axillary drainage, visualized after subdermal or periareolar injections.^1,2,5,9)^ Tumors located in deeper portions of the breast may preferentially follow this pathway and drain directly into the interpectoral or internal mammary nodes.^10)^ In our case, the primary tumor was situated deep within the breast, which likely contributed to isolated interpectoral node involvement.

Imaging findings also highlighted a limitation of SN mapping; although MRI clearly demonstrated an enhancing interpectoral node, it was not identified on lymphoscintigraphy or dye mapping. This discrepancy may be related to both tumor-related partial replacement of the nodal parenchyma, which can impair tracer uptake^3,5)^—and, importantly, the distinct deep lymphatic drainage pattern not reached by superficial injections.^1,2,9)^

Such anatomical variations can affect the accuracy of SN mapping, and the non-visualization observed in our case is considered a representative example of this phenomenon. The present case illustrates a typical limitation of conventional SN mapping.

Given these factors, the absence of tracer uptake in our patient is more plausibly explained by the anatomical differences in lymphatic drainage between the axillary and interpectoral systems than by impaired phagocytic function alone. These observations suggest that the interpectoral node may represent a potential “blind spot” of routine SN mapping,^1,2,5,9)^ and that preoperative cross-sectional imaging—particularly MRI—plays a crucial complementary role in identifying alternative lymphatic pathways and preventing understaging. Although the final pathological stage was pN1a, PMRT was omitted in accordance with the National Comprehensive Cancer Network and Japanese Breast Cancer Society guidelines, which allow omission of selected patients with only 1 positive node and favorable biological features. In our case, the tumor showed ER/PgR positivity, HER2 negativity, low Ki-67 expression, and absence of lymphovascular invasion, placing the patient in a low-risk subgroup. The patient remained disease-free for 54 months, supporting the omission of PMRT and chemotherapy. This favorable clinical course further reinforces that the treatment strategy is consistent with the current guideline-based recommendations for low-risk pN1a disease.

Taken together, historical observations, contemporary series, and rare case reports, including the present case, indicate that isolated interpectoral node metastases are possible, although they are exceedingly rare. Recognition of this pattern, combined with meticulous imaging and pathological assessments, is essential to ensure accurate staging and optimal management.

## CONCLUSIONS

Our case study demonstrates that interpectoral nodes occasionally evade SN mapping. Although isolated interpectoral node metastasis is extraordinarily rare, awareness of this phenomenon, together with careful preoperative MRI, staging ultrasound, and thorough pathological assessment, including extranodal extension, is crucial to avoid understaging and ensure appropriate treatment planning.

## DECLARATIONS

### Availability of data and materials

All data generated or analyzed are included in this article.
